# Triglyceride Glucose Index Is More Closely Related to Hyperuricemia Than Obesity Indices in the Medical Checkup Population in Xinjiang, China

**DOI:** 10.3389/fendo.2022.861760

**Published:** 2022-03-02

**Authors:** Mayina Kahaer, Bei Zhang, Wujin Chen, Meiting Liang, Yi He, Miao Chen, Rui Li, Tingting Tian, Cheng Hu, Yuping Sun

**Affiliations:** ^1^ School of Public Health, Xinjiang Medical University, Urumqi, China; ^2^ Department of Microbiology, School of Basic Medical Sciences, Xinjiang Medical University, Urumqi, China; ^3^ Department of Rheumatology and Immunology, Shanghai Jiao Tong University Affiliated Sixth People’s Hospital, Shanghai, China; ^4^ Shanghai Key Laboratory of Diabetes Mellitus, Shanghai Clinical Center for Diabetes, Shanghai Diabetes Institute, Shanghai Jiao Tong University Affiliated Sixth People’s Hospital, Shanghai, China; ^5^ Department of Endocrinology and Metabolism, Fengxian Central Hospital Affiliated to the Southern Medical University, Shanghai, China

**Keywords:** Triglyceride glucose index, serum uric acid, obesity indices, hyperuricemia, medical checkup population

## Abstract

**Background:**

Hyperuricemia (HUA) is a metabolic anomaly with an increased incidence rate, causing a global medical burden. Several studies have confirmed that obesity and insulin resistance (IR) are the risk factors for HUA. Reports on the predictive power of different obesity indices for HUA are limited. This study aimed to compare the association between different general, abdominal, and visceral obesity indices and markers of the IR-triglyceride glucose (TyG) index with serum uric acid (SUA) and to assess the ability of these indices to predict HUA.

**Methods:**

A total of 2243 participants were recruited from Barkol County Hospital and surrounding township hospitals in Xinjiang. Obesity indices, including the atherogenic index of plasma, cardiometabolic index, visceral adiposity index, lipid accumulation product index, a body shape index, body roundness index, waist circumference, waist-to-height ratio, body mass index, and TyG index, were divided into four quartiles. Moreover, partial correlations and logistic regression were used to analyze the association between these indices and SUA. The area under the curve (AUC) and receiver operating characteristic curves were used to analyze the predictive value of these indices for HUA.

**Results:**

After controlling for confounding variables, the association between the TyG index and HUA was stronger than that between the obesity indices in both males and females. The odds ratios (ORs) for HUA in the highest quartile of the TyG index were 2.098 (95% confidence interval, 1.555–2.831) in males and 7.788 (95% CI, 3.581–16.937) in females. For males, the AIP, CMI, VAI, LAP index, and TyG index were able to discriminate HUA, and the TyG index showed the highest AUC value of 0.586 (95% CI, 0.557–0.614; *P* < 0.001). For females, all indices, except BMI, can discriminate HUA. Moreover, the visceral obesity index CMI showed the highest AUC value of 0.737 (95% CI, 0.691–0.782; *P* < 0.001). Meanwhile, the TyG index had a relatively high AUC value of 0.728 (95% CI, 0.682–0.773; *P* < 0.001).

**Conclusion:**

The TyG index was significantly related to HUA and was superior to obesity indices in identifying HUA in the medical checkup population in Xinjiang, China.

## Introduction

Uric acid is the final product of purine metabolism, and hyperuricemia (HUA) is caused by the excessive production or insufficient excretion of uric acid in the body, the incidence rate of which is increasing annually worldwide. Data from a national survey have demonstrated that the prevalence of HUA among Chinese adults during 2010–2014 was 13.3% (1). According to the National Health and Nutrition Examination Survey, approximately 21% of American adults have HUA (2). The age-standardized prevalence of HUA in the general Korean population is 11.4% (3). Simultaneously, a number of epidemiological studies have reported that HUA causes gout and increases the risk of ischemic stroke, acute myocardial infarction, and other cardiovascular events (4, 5).

Both obesity and insulin resistance (IR) are associated with HUA (6, 7). The general obesity index body mass index (BMI) and abdominal obesity indices waist circumference (WC) and waist-to-height ratio (WHtR) have some implications in predicting the incidence of HUA. However, these indices cannot clearly distinguish between visceral and subcutaneous fats. Previous studies have confirmed a positive correlation between visceral fat deposition and increased uric acid production (8). Since the direct estimation of visceral fat requires diagnostic imaging, which is expensive and has low epidemiological availability, there has been increasing interest in finding simple and effective alternative markers of visceral obesity. In recent years, some lipid and visceral obesity-related indices, such as visceral adiposity index (VAI), lipid accumulation product (LAP) index, atherogenic index of plasma (AIP), cardiometabolic index (CMI), a body shape index (ABSI), and body roundness index (BRI), have also been proposed as supplementary indices to assess obesity and to predict the incidence of related metabolic diseases. The triglyceride glucose (TYG) index is a marker of IR and is associated with uric acid through obesity (9). A comparison with the gold standard method revealed that the TyG index was more suitable for the determination of IR than alternative indices, such as the homeostasis model assessment-estimated insulin resistance.

Many studies have investigated these indices in relation to cardiovascular diseases and diabetes, but few have predicted HUA (10, 11). Simultaneously, although many studies have pointed out that obesity indices can predict the risk of HUA, there was no conclusion on which indices were more suitable for predicting the risk of HUA in Xinjiang population, China. Therefore, this study aimed to use a cross-sectional survey to analyze and compare six visceral obesity indices (AIP, CMI, VAI, LAP index, ABSI, and BRI) with the general obesity index BMI, abdominal obesity indices WC and WHtR, and TyG index to predict the risk of HUA, determine more suitable risk predictors for HUA in our population, and provide a basis for early prevention of HUA.

## Methods

### Study Participants

This was a cross-sectional, observational study. All participants were selected from the medical checkup population of Barkol County Hospital and the surrounding township hospitals from May 2016 to May 2021. A total of 2243 participants (age range, 20–68 years) were included in this study. Individuals with any of the following conditions were excluded: (1) chronic kidney disease or renal function impairment, (2) long-term use of uric acid-lowering drugs, (3) malignant tumors, and (4) autoimmune diseases that may affect SUA levels. Trained research interviewers administered standardized questionnaires through face-to-face interviews. The questionnaire included age, sex, medication history, and medical history. Height, weight, and WC were measured using standard methods. Blood pressure was measured using an electronic sphygmomanometer. Fasting blood samples (5 mL) were drawn from the participants under strict aseptic conditions and the serum was separated by centrifugation at 3000 rpm for 15 min after clotting. Myriad BS-800M fully automated biochemical analyzer and its matching reagents were used to assess the level of serum uric acid (SUA), fasting plasma glucose(FPG), high-density lipoprotein cholesterol (HDL-C), low-density lipoprotein cholesterol (LDL-C), total cholesterol (TC), triacylglycerol (TG), blood urea nitrogen (BUN), and creatinine (Cre) in Barkol County Hospital.

### Definition and Obesity Index Calculations

HUA was defined as an SUA level >7.0 mg/dL according to the guidelines for the diagnosis and management of HUA and gout in China (2019) (12). According to previous studies, obesity indices were calculated using the following formula (13, 14):


$$\text{BMI}={\text{weight (kg)/height}}^{2}({\text{m}}^{2})$$

AIP=log (TG [mmol/L]/HDL−C [mmol/L])

CMI=TG/HDL−C×WHtR

$$\begin{array}{c} \text{VAI (males)}=(\text{WC [cm]}/39.68+[1.88\times \text{BMI]})\times (\text{TG [mmol/L]/}1.03)\times (1.31\text{/HDL}-\text{C [mmol/L]})\text{ VAI (females)}\\ \;=(\text{WC [cm]}/36.58+[1.89\times \text{BMI]})\times (\text{TG [mmol/L]/}0.81)\times \left(1.52\text{/HDL}-\text{C [mmol/L]}\right) \end{array}$$

LAP index (females)=TG (mmol/L)×(WC [cm]−58)

LAP index (males)=TG (mmol/L)×(WC [cm]−65)

TyG index=log (TG[mmol/L]× fasting plasma glucose (FPG)[mmol/L]/2)

$$\text{ABSI}={\text{WC (cm)/(height [cm])}}^{1/2}\times {\left({\text{BMI}}^{2}\right)}^{1/3}$$

$$\text{BRI}=364.2-365.5\times {\left[1-(\text{WC/}2\pi )/(0.5\times \text{height})\right]}^{2}{]}^{1/2}$$

### Statistical Analyses

The measures of normal distribution are expressed as means ± standard deviation, and the two groups were compared using an independent sample t-test. One-way analysis of variance was used to compare more than two groups. Non-normally distributed measures are expressed as M (P25, P75), the Mann–Whitney U-test was used for comparison between two groups, and the Kruskal–Wallis H-test was used for comparison between more than two groups. Partial correlation analysis was performed to assess the correlation between the different indices and SUA. Logistic analysis was performed to analyze the association between the different indices and HUA. A receiver operating characteristic (ROC) curve was used to analyze the predictive value of the different indices for HUA. All statistical analyses were performed using the Statistical Package for the Social Sciences version 21.0 (International Business Machines Corporation) and GraphPad Prism version 6.0. Statistical significance was defined as a two-tailed *P*-value < 0.05.

## Results

A total of 2243 participants (1616 males and 627 females) were enrolled in the study, with an age range of 20–69 years (mean age, 41.55 ± 12.70 years). The basic characteristics of all participants are summarized in **Table 1**. Participants with HUA were older than those without HUA. Compared to participants with normal SUA, participants with HUA had significantly higher WC; BMI; WHtR; systolic blood pressure (SBP); diastolic blood pressure (DBP); TG, LDL-C, BUN, and Cre levels; AIP; CMI; VAI; LAP index; TyG index; ABSI; and BRI (all *P* < 0.05). No significant differences were found in FPG, TC, and HDL-C levels between the two groups.

**Table 1:** Clinical characteristics of study participants with and without hyperuricemia.

Variables	Non-hyperuricemia (n = 1471)	Hyperuricemia (n = 772)	χ²/t/z	*P*-value
Males, n (%)	1001 (68.05)	615 (79.66)	33.91	<0.001
Age (years)	42.44 ± 12.24	45.17 ± 12.28	−5.014	<0.001
SBP (mmHg)	121.36 ± 17.83	123.14 ± 17.87	−2.252	0.024
DBP (mmHg)	78.95 ± 11.59	80.65 ± 13.33	−3.129	0.002
SUA (mg/dL)	4.72 ± 1.28	8.20 ± 1.29	−60.870	<0.001
FPG (mmol/L)	5.13 ± 1.38	5.13 ± 1.80	−0.020	0.984
TC (mmol/L)	4.50 ± 1.84	4.44 ± 1.56	0.749	0.454
TG (mmol/L)	1.28 (0.86–2.1)	1.85 (1.2–3.18)	−11.603	<0.001
HDL-C (mmol/L)	1.19 ± 0.51	1.17 ± 0.41	0.491	0.623
LDL-C (mmol/L)	2.67 (2.1–3.33)	2.87 (2.3–3.49)	−4.030	<0.001
BUN (mmol/L)	5.33 ± 3.32	5.73 ± 3.09	−2.767	0.006
Cre (mL/min)	80.38 ± 41.72	86.08 ± 34.17	−3.261	0.001
TyG index	8.61 ± 0.73	8.96 ± 0.71	−10.802	<0.001
General obesity index
BMI (kg/m^2^)	25.01 (22.65–27.68)	25.51 (23.18–27.85)	−2.739	0.017
Abdominal obesity indices
WC (cm)	89.48 ± 12.89	91.44 ± 13.70	−3.339	0.001
WHtR	0.53 ± 0.08	0.54 ± 0.09	−3.022	0.003
Visceral obesity indices
AIP	0.09 ± 0.34	0.24 ± 0.34	−9.887	<0.001
CMI	0.6 (0.36–1.18)	0.9 (0.54–1.72)	−9.792	<0.001
VAI	1.72 (1.07–3.1)	2.38 (1.44–4.42)	−8.387	<0.001
LAP index	32.19 (18.48–59.8)	46.2 (27.39–87.48)	−9.146	<0.001
ABSI	0.80 ± 0.07	0.81 ± 0.09	−2.977	0.003
BRI	4.11 ± 1.57	4.34 ± 1.92	−3.073	0.002

SBP, systolic blood pressure; DBP, diastolic blood pressure; SUA, serum uric acid; FPG, fasting plasma glucose; TC, total cholesterol; TGs, triglycerides; HDL-C, high-density lipoprotein cholesterol; LDL-C, low-density lipoprotein cholesterol; BUN, blood urea nitrogen; Cre, creatinine; TyG index, triglyceride glucose index; BMI, body mass index; WC, waist circumference; WHtR, waist-to-height ratio; AIP, atherogenic index of plasma; CMI, cardiometabolic index; VAI, visceral adiposity index; LAP index, lipid accumulation product index; ABSI, a body shape index; BRI, body roundness index.

The partial correlation coefficients between the different indices and SUA are shown in **Table 2**. SUA levels were significantly correlated with the TyG index, AIP, CMI, VAI, and LAP index after adjusting for age in both males and females (all *P* < 0.05). Among them, the SUA level had the strongest positive correlation with the TyG index in all participants (r = 0.332, 0.229, and 0.4, respectively; all *P* < 0.05), whereas the CMI, VAI, and LAP index had a relatively high correlation. SUA levels were significantly correlated with WC, WHtR, ABSI, and BRI in females only.

**Table 2:** Partial correlation coefficients between different indices and SUA.

Variables	Total		Males		Females	
	*r*	*P*-value	*r*	*P*-value	*r*	*P*-value
TyG index	0.332	<0.001	0.229	<0.001	0.4	<0.001
BMI	0.049	0.021	0.014	0.563	0.051	0.2
WC	0.118	<0.001	0.029	0.247	0.199	<0.001
WHtR	0.079	<0.001	0.034	0.178	0.188	<0.001
AIP	0.31	<0.001	0.198	<0.001	0.41	<0.001
CMI	0.226	<0.001	0.151	<0.001	0.321	<0.001
VAI	0.198	<0.001	0.152	<0.001	0.316	<0.001
LAP index	0.198	<0.001	0.126	<0.001	0.331	<0.001
ABSI	0.081	<0.001	0.014	0.573	0.243	<0.001
BRI	0.055	0.009	0.018	0.467	0.161	<0.001

TyG index, triglyceride glucose index; BMI, body mass index; WC, waist circumference; WHtR, waist-to-height ratio; AIP, atherogenic index of plasma; CMI, cardiometabolic index; VAI, visceral adiposity index; LAP index, lipid accumulation product index; ABSI, a body shape index; BRI, body roundness index.

Multivariate logistic regression revealed the odds ratios (ORs) and 95% confidence intervals (CIs) for HUA according to sex-specific TyG index, BMI, WC, WHtR, AIP, CMI, VAI, LAP index, ABSI, and BRI quartiles. After full adjustment for age, SBP, DBP, and selected biochemical indices in model 3, compared with the first quartile, the other three quartiles of visceral obesity TyG index were strongly associated with HUA in both males and females (all *P* < 0.05). For males and females, the ORs for HUA in the upper quartile of the TyG index were 2.098 (95% CI, 1.555–2.831) and 7.788 (95% CI, 3.581–16.937), respectively. The adjusted relative risk of HUA increased with increasing TyG quartiles. The results are presented in **Tables 3**, **4**.

**Table 3:** Multivariate logistic regression of different indices for HUA (males).

Variables	Quartile 1	Quartile 2		Quartile 3		Quartile 4	
		OR (95% CI)	*P*-value	OR (95% CI)	*P*-value	OR (95% CI)	*P*-value
TyG index	≤8.33	8.34–8.78		8.79–9.34		≥9.35	
Model 1	Reference	1.573 (1.169–2.116)	0.003	1.818 (1.355–2.441)	<0.001	2.202 (1.643–2.952)	<0.001
Model 2	Reference	1.519 (1.127–2.047)	0.006	1.748 (1.3–2.351)	<0.001	2.131 (1.585–2.867)	<0.001
Model 3	Reference	1.501 (1.108–2.032)	0.009	1.762 (1.303–2.383)	<0.001	2.098 (1.555–2.831)	<0.001
BMI	≤23	24–25		26–28		≥29	
Model 1	Reference	1.277 (0.959–1.7)	0.094	1.452 (1.089–1.935)	0.011	1.15 (0.861–1.535)	0.343
Model 2	Reference	1.221 (0.911–1.635)	0.182	1.351 (1.003–1.82)	0.048	1.029 (0.757–1.4)	0.855
Model 3	Reference	1.211 (0.901–1.629)	0.205	1.369 (1.013–1.85)	0.041	1.045 (0.767–1.424)	0.781
WC	≤82	83–90		91–99		≥100	
Model 1	Reference	1.164 (0.88–1.539)	0.287	1.189 (0.893–1.584)	0.236	1.04 (0.78–1.387)	0.79
Model 2	Reference	1.116 (0.839–1.483)	0.451	1.077 (0.8–1.45)	0.626	0.914 (0.673–1.241)	0.564
Model 3	Reference	1.128 (0.846–1.506)	0.412	1.091 (0.807–1.474)	0.573	0.941 (0.691–1.281)	0.697
WHtR	≤0.49	0.5–0.53		0.54–0.59		≥0.6	
Model 1	Reference	1.1 (0.827–1.463)	0.513	1.195 (0.9–1.588)	0.219	1.055 (0.792–1.404)	0.715
Model 2	Reference	1.042 (0.779–1.394)	0.78	1.093 (0.814–1.468)	0.555	0.927 (0.683–1.258)	0.627
Model 3	Reference	1.03 (0.767–1.382)	0.845	1.109 (0.823–1.495)	0.496	0.945 (0.695–1.285)	0.717
AIP	≤−0.04	−0.03–0.18		0.19–0.45		≥0.46	
Model 1	Reference	1.475 (1.102–1.974)	0.009	1.531 (1.145–2.048)	0.004	1.869 (1.399–2.497)	<0.001
Model 2	Reference	1.444 (1.077–1.938)	0.014	1.5 (1.119–2.011)	0.007	1.819 (1.355–2.444)	<0.001
Model 3	Reference	1.418 (1.054–1.908)	0.021	1.502 (1.116–2.02)	0.007	1.839 (1.364–2.48)	<0.001
CMI	≤0.47	0.48–0.8		0.81–1.54		≥1.55	
Model 1	Reference	1.454 (1.083–1.953)	0.013	1.896 (1.417–2.536)	<0.001	1.808 (1.35–2.421)	<0.001
Model 2	Reference	1.407 (1.045–1.895)	0.024	1.85 (1.379–2.483)	<0.001	1.739 (1.289–2.346)	<0.001
Model 3	Reference	1.39 (1.029–1.877)	0.032	1.854 (1.376–2.499)	<0.001	1.752 (1.292–2.374)	<0.001
VAI	≤1.19	1.2–1.98		1.99–3.74		≥3.75	
Model 1	Reference	1.383 (1.031–1.855)	0.031	1.883 (1.409–2.516)	<0.001	1.668 (1.246–2.231)	0.001
Model 2	Reference	1.349 (1.003–1.814)	0.048	1.82 (1.358–2.44)	<0.001	1.608 (1.194–2.165)	0.002
Model 3	Reference	1.33 (0.986–1.794)	0.062	1.822 (1.354–2.453)	<0.001	1.615 (1.194–2.186)	0.002
LAP index	≤21.6	21.61–39.96		39.97–77.14		≥77.15	
Model 1	Reference	1.821 (1.356–2.445)	<0.001	1.758 (1.309–2.362)	<0.001	2.091 (1.56–2.803)	<0.001
Model 2	Reference	1.761 (1.303–2.38)	<0.001	1.709 (1.261–2.318)	0.001	2.016 (1.48–2.747)	<0.001
Model 3	Reference	1.787 (1.317–2.425)	<0.001	1.749 (1.283–2.383)	<0.001	2.043 (1.493–2.794)	<0.001
ABSI	≤0.76	0.77–0.8		0.81–0.84		≥0.85	
Model 1	Reference	0.895 (0.669–1.197)	0.454	1.351 (1.021–1.788)	0.035	1.351 (1.012–1.788)	0.827
Model 2	Reference	0.872 (0.651–1.168)	0.359	1.291 (0.973–1.713)	0.077	0.968 (0.722–1.296)	0.825
Model 3	Reference	0.867 (0.645–1.166)	0.346	1.3 (0.976–1.73)	0.073	0.981 (0.729–1.319)	0.899
BRI	≤3.08	3.09–3.93		3.94–5.11		≥5.12	
Model 1	Reference	1.1 (0.827–1.463)	0.513	1.195 (0.9–1.588)	0.219	1.055 (0.792–1.404)	0.715
Model 2	Reference	1.042 (0.779–1.394)	0.78	1.093 (0.814–1.468)	0.555	0.927 (0.683–1.258)	0.627
Model 3	Reference	1.03 (0.767–1.382)	0.845	1.109 (0.823–1.495)	0.496	0.945 (0.695–1.285)	0.717

Model 1: unadjusted; model 2: adjusted for age, SBP, and DBP; model 3: adjusted for all variables in model 2 plus BUN, Cre, TC, and LDL-C. TyG index, triglyceride glucose index; BMI, body mass index; WC, waist circumference; WHtR, waist-to-height ratio; AIP, atherogenic index of plasma; CMI, cardiometabolic index; VAI, visceral adiposity index; LAP index, lipid accumulation product index; ABSI, a body shape index; BRI, body roundness index.

**Table 4:** Multivariate logistic regression of different indices for HUA (females).

Variables	Quartile 1	Quartile 2		Quartile 3		Quartile 4	
		OR (95% CI)	*P*-value	OR (95% CI)	*P*-value	OR (95% CI)	*P*-value
TyG index	≤7.99	8–8.36		8.37–8.81		≥8.82	
Model 1	Reference	3.619 (1.759–7.443)	<0.001	3.665 (1.787–7.52)	<0.001	12.923 (6.487–25.745)	<0.001
Model 2	Reference	3.622 (1.756–7.472)	<0.001	3.718 (1.809–7.64)	<0.001	12.834 (6.434–25.603)	<0.001
Model 3	Reference	3.125 (1.373–7.114)	0.007	2.9 (1.275–6.593)	0.011	7.788 (3.581–16.937)	<0.001
BMI	≤22	23–25		26–27		≥28	
Model 1	Reference	0.657 (0.383-1.125)	0.126	0.674 (0.395–1.149)	0.148	1.627 (1.004–2.634)	0.048
Model 2	Reference	0.593 (0.342–1.028)	0.063	0.544 (0.309–0.956)	0.034	1.195 (0.698–2.047)	0.516
Model 3	Reference	0.528 (0.256–1.092)	0.085	0.884 (0.449–1.74)	0.721	1.192 (0.611–2.325)	0.608
WC	≤79	80–87		88–94		≥95	
Model 1	Reference	2.125 (1.224–3.688)	0.007	1.729 (0.991–3.015)	0.054	2.547 (1.473–4.407)	0.001
Model 2	Reference	1.846 (1.045–3.261)	0.035	1.525 (0.852–2.729)	0.155	1.946 (1.071–3.534)	0.029
Model 3	Reference	1.327 (0.647–2.722)	0.44	1.722(0.853–3.475)	0.129	1.727 (0.837–3.564)	0.139
WHtR	≤0.49	0.5–0.53		0.54–0.57		≥0.58	
Model 1	Reference	1.992 (1.146–3.462)	0.015	1.527 (0.864–2.699)	0.145	2.768 (1.614–4.748)	<0.001
Model 2	Reference	1.808 (1.028–3.179)	0.04	1.21 (0.661–2.212)	0.537	2.094 (1.155–3.798)	0.015
Model 3	Reference	1.375 (0.672–2.813)	0.383	1.52(0.727–3.177)	0.266	1.88 (0.902–3.919)	0.092
AIP	≤−0.22	−0.21 to −0.22		−0.01–0.19		≥0.2	
Model 1	Reference	2.089 (1.051–4.155)	0.036	3.887 (2.026–7.457)	<0.001	9.093 (4.828–17.125)	<0.001
Model 2	Reference	1.924 (0.958–3.863)	0.066	3.611 (1.868–6.983)	<0.001	8.004 (4.214–15.202)	<0.001
Model 3	Reference	1.751 (0.709–4.323)	0.224	4.815 (2.086–11.117)	<0.001	9.127 (4.008–20.784)	<0.001
CMI	≤0.32	0.33–0.49		0.5–0.85		≥0.86	
Model 1	Reference	2.107 (1.059–4.191)	0.034	3.212 (1.661–6.209)	0.001	10.143 (5.389–19.09)	<0.001
Model 2	Reference	1.909 (0.953–3.825)	0.068	2.833 (1.452–5.527)	0.002	8.708 (4.576–16.572)	<0.001
Model 3	Reference	1.351 (0.551–3.31)	0.511	3.52 (1.552–7.983)	0.003	8.839 (3.976–19.654)	<0.001
VAI	≤1.15	1.16–1.81		1.82–3.08		≥3.09	
Model 1	Reference	2.413 (1.225–4.753)	0.011	3.376 (1.749–6.515)	<0.001	9.218 (4.897–17.35)	<0.001
Model 2	Reference	2.207 (1.111–4.383)	0.024	3.139 (1.614–6.107)	0.001	8.065 (4.247–15.313)	<0.001
Model 3	Reference	2.278 (0.948–5.472)	0.066	3.834 (1.645–8.934)	0.002	9.34 (4.119–21.18)	<0.001
LAP index	≤20.28	20.29–30.8		30.81–51.7		≥51.71	
Model 1	Reference	1.755 (0.926–3.325)	0.085	2.46 (1.335–4.533)	0.004	6.89 (3.847–12.339)	<0.001
Model 2	Reference	1.567 (0.814–3.015)	0.179	2.181 (1.159–4.103)	0.016	5.792 (3.126–10.729)	<0.001
Model 3	Reference	1.123 (0.492–2.563)	0.782	2.579 (1.214–5.48)	0.014	4.843 (2.294–10.225)	<0.001
ABSI	≤0.76	0.77–0.79		0.8–0.83		≥0.84	
Model 1	Reference	1.104 (0.663–1.838)	0.704	1.234 (0.699–2.178)	0.468	1.84 (1.106–3.061)	0.019
Model 2	Reference	1.034 (0.615–1.737)	0.9	1.167 (0.653–2.085)	0.602	1.638 (0.965–2.782)	0.068
Model 3	Reference	1.409 (0.747–2.658)	0.29	1.054 (0.492–2.259)	0.893	1.476 (0.764–2.852)	0.247
BRI	≤3.12	3.13–4.03		4.04–4.82		≥4.83	
Model 1	Reference	1.992 (1.146–3.462)	0.015	1.527 (0.864–2.699)	0.145	2.768 (1.614–4.748)	<0.001
Model 2	Reference	1.808 (1.028–3.179)	0.04	1.21 (0.661–2.212)	0.537	2.094 (1.155–3.798)	0.015
Model 3	Reference	1.375 (0.672–2.813)	0.383	1.52 (0.727–3.177)	0.266	1.88 (0.902–3.919)	0.092

Model 1: unadjusted; model 2: adjusted for age, SBP, and DBP; model 3: adjusted for all variables in model 2 plus BUN, Cre, TC, and LDL-C. TyG index, triglyceride glucose index; BMI, body mass index; WC, waist circumference; WHtR, waist-to-height ratio; AIP, atherogenic index of plasma; CMI, cardiometabolic index; VAI, visceral adiposity index; LAP index, lipid accumulation product index; ABSI, a body shape index; BRI, body roundness index.

**Figure 1:**
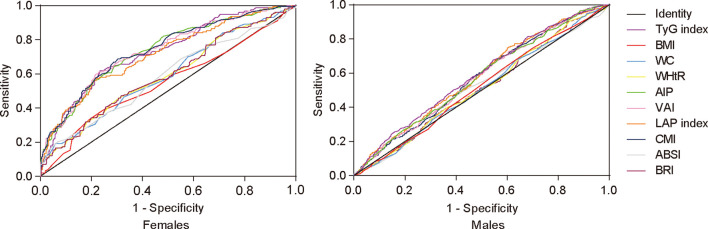
Receiver operating characteristic curve analysis by sex. TyG index, triglyceride glucose index; BMI, body mass index; WC, waist circumference; WHtR, waist-to-height ratio; AIP, atherogenic index of plasma; CMI, cardiometabolic index; VAI, visceral adiposity index; LAP index, lipid accumulation product index; ABSI, a body shape index; BRI, body roundness index.

The ROC curves of the different indices for HUA are presented in **Table 5** and **Figure 1**. For males, the TyG index, AIP, CMI, VAI, and LAP index were able to discriminate HUA, and the TyG index showed the highest area under the curve (AUC) value of 0.586 (95% CI, 0.557–0.614; *P* < 0.05), with a cutoff value of 8.353 according to the maximum Youden index of 0.133. For females, all indices, except BMI, could discriminate HUA. The CMI showed the highest AUC value of 0.737 (95% CI, 0.691–0.782; *P* < 0.05), with a cutoff value of 0.595 according to the maximum Youden index of 0.384. Meanwhile, the TyG index, AIP, VAI, and LAP index had a relatively high AUC value.

**Table 5:** Comparison of the ability of different indices to predict HUA.

Variable	AUC (95% CI)	Cut-off	Sensitivity	Specificity	Youden index	*P*-value
Males						
BMI	0.515 (0.487–0.544)	24.07	0.759	0.293	0.052	0.295
WC	0.507 (0.478–0.536)	86.5	0.675	0.369	0.043	0.638
WHtR	0.513 (0.484–0.541)	0.433	0.948	0.089	0.037	0.389
AIP	0.569 (0.54–0.597)	0.121	0.636	0.486	0.122	<0.001
TyG index	0.586 (0.557–0.614)	8.353	0.815	0.318	0.133	<0.001
CMI	0.569 (0.541–0.597)	0.605	0.701	0.422	0.123	<0.001
VAI	0.568 (0.54–0.596)	1.444	0.728	0.392	0.12	<0.001
LAP index	0.578 (0.55–0.606)	27.1	0.748	0.399	0.147	<0.001
ABSI	0.514 (0.485–0.543)	0.803	0.514	0.547	0.061	0.343
BRI	0.513 (0.484–0.541)	2.613	0.901	0.166	0.067	0.389
Females						
BMI	0.55 (0.495–0.604)	27.291	0.363	0.8	0.163	0.063
WC	0.59 (0.538–0.642)	88.5	0.465	0.672	0.137	0.001
WHtR	0.591 (0.539–0.644)	0.452	0.904	0.147	0.051	0.001
AIP	0.734 (0.688–0.779)	0.029	0.688	0.672	0.36	<0.001
TyG index	0.728 (0.682–0.773)	8.575	0.631	0.743	0.373	<0.001
CMI	0.737 (0.691–0.782)	0.595	0.688	0.696	0.384	<0.001
VAI	0.735 (0.689–0.78)	2.196	0.662	0.704	0.367	<0.001
LAP index	0.715 (0.668–0.762)	45.04	0.573	0.781	0.354	<0.001
ABSI	0.579 (0.525–0.632)	0.788	0.682	0.464	0.145	0.003
BRI	0.591 (0.539–0.644)	4.233	0.471	0.683	0.154	0.001

TyG index, triglyceride glucose index; BMI, body mass index; WC, waist circumference; WHtR, waist-to-height ratio; AIP, atherogenic index of plasma; CMI, cardiometabolic index; VAI, visceral adiposity index; LAP index, lipid accumulation product index; ABSI, a body shape index; BRI, body roundness index.

## Discussion

### Main Findings

This study investigated and compared the predictive strength of the TyG index and nine obesity indices reflecting general (BMI), abdominal (WC, WHtR), and visceral (AIP, CMI, VAI, LAP index, ABSI, BRI) obesity in the assessment of HUA in a medical checkup population in Xinjiang, China. The main findings of this study were as follows: (1) the TyG index was significantly associated with HUA, and the association remained significant after controlling for age, SBP, DBP, and selected biochemical indices. Further stratification showed that when the TyG index was higher (in the third and fourth quartiles), the risk of developing HUA was higher (2). Some visceral obesity indices were also associated with HUA, but were inferior to the TyG index (3). The TyG index was a better predictor of HUA, especially in females. Therefore, the TyG index may be the best choice for the HUA risk screening index for this regional population.

### General and Abdominal Obesity Indices With Hyperuricemia (HUA)

In many previous studies, obesity was often reflected in BMI, WC, and WHtR, and some studies have shown that these indices are associated with HUA (15). However, BMI reflects only the degree of obesity in humans. Although WC and WHtR can reflect abdominal obesity, they cannot distinguish subcutaneous fat from visceral fat (16, 17), and the accumulation of visceral fat is more likely to lead to disorders of uric acid metabolism than subcutaneous fat (18). However, BMI, WC, and WHtR were not significantly associated with HUA in the present study. The general obesity indices for this population may not be the most appropriate way to assess HUA, which is characterized by excessive weight and fat accumulation. There were differences in body fat distribution between the ethnic groups (19). According to the World Health Organization guidelines, thresholds for BMI, WC, and WHtR are recommended for several different ethnicities and populations (20).

### Visceral Obesity Indices With HUA

Studies have shown that obesity, especially visceral obesity, is closely associated with HUA. However, there is no definitive conclusion regarding which criterion is more valuable in reflecting visceral fat content and predicting HUA. One of the important reasons for this is that there are some limitations to the various criteria for measuring obesity. New indices, such as the ABSI and BRI, have been presented to distribute adipose tissue more precisely, and some studies have reported a close association between these indices and HUA (21, 22). In contrast to previous studies, no correlation between these indices and HUA was found in the present study. The reasons for this different conclusion may include the following: (1) participants from different areas had different lifestyles and variations in age distribution and sex composition. (2) The ABSI and BRI cannot distinguish between the distribution of subcutaneous and visceral adipose tissues (23). (3) The ABSI was originally established to predict mortality in a follow-up cohort (24), whereas this study utilized it in a cross-sectional manner. This may be the reason for the low screening value.

Previous studies have shown that indices consisting of TG were well suited to identify individuals with unhealthy metabolism (25). The CMI is a new index for evaluating visceral obesity using lipid parameters TG and HDL-C to waist height ratio, which has been shown to be associated with several metabolic syndromes (26, 27). The VAI integrates the traditional obesity indices WC and BMI with TG and HDL-C, which can better indicate visceral fat content and its distribution (28). The LAP index integrates WC, an indicator of abdominal obesity, and TG, which is closely related to visceral fat distribution (6). The present study showed that the CMI, VAI, and LAP index could affect uric acid levels and increase the risk of HUA, which is consistent with the results of previous studies (29). Compared with traditional anthropometric indices, the CMI, VAI, and LAP index have better predictive power for HUA in both males and females. In particular, the sensitivity of the ROC curve was higher in females than in males. This may be related to the fact that the CMI, LAP index, and VAI fully integrate WC, an indicator for assessing abdominal obesity, and TG, which is related to the visceral fat distribution. This suggests that higher visceral fat accumulation expressed by elevated LAP has a greater effect on uric acid metabolism than BMI values, which includes less specificity for subcutaneous fat accumulation. Visceral fat promotes the synthesis of phosphoribosyl pyrophosphate from very low-density lipoprotein and ribose 5-phosphate, which leads to the excessive production of uric acid. Meanwhile, the VAI was a better predictor of HUA than BMI, WC, and WHR independently in the Chinese population (30), probably because of the accumulation of visceral fat, which allows free fatty acids (FFAs) to enter the liver *via* the portal vein, increasing the synthesis of TGs and causing hypertriglyceridemia. Moreover, FFAs can increase the synthesis of purines *via* the pentose phosphate pathway. The correlation between the CMI, VAI, and LAP index and uric acid in this study further confirmed the association between visceral fat and HUA.

The AIP is a sensitive index of lipid metabolism disorders proposed by Dobiasova et al. (31), which uses log (TG/HDL-C) as an index that can be used as a predictor of the risk of plasma atherosclerosis development, and its value is negatively correlated with LDL particle size and LDL-C as a routine index of clinical testing if abnormally elevated, leading to a high risk of cardiovascular disease. The AIP has been significantly associated with HUA, hypercholesterolemia, hyperlipidemia, and metabolic syndrome, which are all risk factors for cardiovascular disease (32). Zhu et al. found a positive association between higher levels of AIP, a new biomarker associated with obesity (33). In the present study, the partial correlation analysis suggested that the AIP was positively associated with SUA and could be used as an independent risk factor for predicting HUA in both sexes. This is consistent with the results of a cross-sectional study by Chang et al. on the association between blood uric acid and AIP in a population from northeastern China (34). The mechanism may be due to the fact that changes in cholesterol and TG can cause disorders of lipid metabolism, whereas the association between dyslipidemia and HUA is bidirectional. On the one hand, due to the long and cold winter in Xinjiang, the local population has a high proportion of high-purine and high-fat diet intake, which easily causes fat accumulation and disorders of lipid and purine metabolism. On the other hand, high concentrations of uric acid can affect lipid peroxidation and LDL cholesterol oxidation, reducing the activity of the corresponding enzymes and decreasing cholesterol catabolism, causing changes in blood lipids (35).

### The Triglyceride Glucose Index With HUA

Epidemiological studies have established a significant association between IR and SUA (36, 37), and compensatory hyperinsulinemia that occurs after IR can reduce uric acid excretion by renal tubular sodium reabsorption and cause HUA (38). Conversely, higher uric acid levels can reduce nitric oxide bioavailability and mitochondrial oxidative stress, leading to IR (39). Therefore, the evaluation of IR status in a normal physical examination population is beneficial not only for uric acid control but also for the prevention of other metabolic diseases. Traditional IR assessment tools, such as the high insulin glucose clamp test and steady-state mode assessment method, cannot be commonly used because of their complicated, invasive, and expensive characteristics. In recent years, several simple IR assessment tools have been developed and used clinically. The TyG index was developed in 2008 by Simental-Mendía et al. (40) and has better sensitivity and specificity compared to the gold standard for IR detection (41). Some studies have reported that the TyG index is associated with atherosclerosis, metabolic syndrome, and type 2 diabetes mellitus (42–44). Mazidi et al. (9) found a correlation between the TyG index and HUA in Caucasian populations. In the present study, logistic regression models revealed a significant correlation between the TyG index and the risk of HUA in both males and females compared to other obesity indices, even after multivariate adjustment. Moreover, this correlation was consistent across all subgroups after performing a stratified analysis of the TyG index, and the risk of HUA was higher in the high quartile than in the low quartile. The TyG index also had a significant value in identifying HUA in both sexes in the ROC analysis compared to other indices (*P* < 0.001), especially in females. This may be due to the fact that estrogen, as a uric acid producing agent, leads to more complex endocrine factors in its metabolism than in males, as well as differences in lipid metabolism between the sexes. This finding is consistent with the results of a cross-sectional study by Shi et al. (45) in northeastern China. One possible mechanism is that IR affects lipid metabolism by decreasing lipoprotein lipase activity and reducing lipocalin production (4). Second, high TG levels are degraded to FFAs, which are transported to other tissues and accelerate adenosine triphosphate breakdown. Abnormal lipid metabolism also impairs the kidney, decreases renal blood flow, reduces urinary uric acid excretion, and increases SUA levels (46).

This study systematically investigated the predictive value of the TyG index and nine obesity indices for HUA and included a comprehensive range of indices. All data in the study were obtained from the same regional population, which reduced the risk of bias in the sample source. However, this study has some limitations. First, it investigated a specific region, Barkol County, which may not best represent the overall status of ethnicity and related diseases in Xinjiang. Second, it has been previously reported that uric acid levels can be affected by diet, but information on dietary patterns, such as dairy and meat intake, was not analyzed in this study population, this may lead to biased results. Third, the cross-sectional survey could not clearly determine the causal association between the risk factors and the incidence of HUA. Systematic large-scale studies are required to further elucidate the changes in uric acid levels and their associated risk factors.

## Conclusions

In conclusion, different indices were differentially related to HUA and had different predictive abilities. In addition to generalized obesity, abdominal obesity and the resulting visceral fat accumulation also need to be considered. The TyG index was more closely related to HUA than the obesity indices in the medical checkup population in Xinjiang, China. Thus, it can be used as an important index for HUA risk screening and population health management.

## Data Availability Statement

The original contributions presented in the study are included in the article/supplementary material. Further inquiries can be directed to the corresponding authors.

## Ethics Statement

The studies involving human participants were reviewed and approved by The Medical Ethics Committee of the First Affiliated Hospital of Xinjiang Medical University. The patients/participants provided their written informed consent to participate in this study.

## Author Contributions

YS and CH contributed to conception and design of the study. BZ, WC, MC, and TT organized the database. ML, YH, RL performed the statistical analysis. MK wrote the first draft of the manuscript. All authors contributed to manuscript revision, read, and approved the submitted version.

## Funding

This study was funded by the National Natural Science Foundation of China (No.81960169,81760169, and 81900795), the Natural Science Foundation of the Xinjiang Uygur Autonomous Region (No. 2019D01C219 and 2021D01C275),the Project of Scientific Research Program for Universities in Xinjiang Uygur Autonomous Region(No.XJEDU2021Y054),the Postgraduate Research Innovation Project in Xinjiang Uygur Autonomous Region(XJ2021G228).

## Conflict of Interest

The authors declare that the research was conducted in the absence of any commercial or financial relationships that could be construed as a potential conflict of interest.

## Publisher’s Note

All claims expressed in this article are solely those of the authors and do not necessarily represent those of their affiliated organizations, or those of the publisher, the editors and the reviewers. Any product that may be evaluated in this article, or claim that may be made by its manufacturer, is not guaranteed or endorsed by the publisher.
